# Endoplasmic reticulum stress promotes inflammation-mediated proteolytic activity at the ocular surface

**DOI:** 10.1038/s41598-020-59237-3

**Published:** 2020-02-10

**Authors:** Ashley M. Woodward, Antonio Di Zazzo, Stefano Bonini, Pablo Argüeso

**Affiliations:** 1 Schepens Eye Research Institute of Massachusetts Eye and Ear, Department of Ophthalmology, Harvard Medical School, Boston, Massachusetts United States; 2 Ophthalmology Complex Unit, Campus Bio-Medico University of Rome, Rome, Italy

**Keywords:** Endoplasmic reticulum, Corneal diseases

## Abstract

A growing body of evidence implicates endoplasmic reticulum (ER) stress in the pathogenesis of chronic inflammatory and autoimmune disorders. Here, we demonstrate that the proinflammatory cytokine TNFα stimulates matrix metalloproteinase 9 (MMP9) at the ocular surface through a c-Fos-dependent mechanism of ER stress. We found positive reactivity of the molecular chaperone BiP/GRP78 in conjunctival epithelium of patients with ocular cicatricial pemphigoid and increased levels of BiP/GRP78, sXBP1 and GRP94 in human corneal epithelial cells treated with TNFα. Pharmacological blockade of ER stress *in vitro* using dexamethasone or the chemical chaperones TUDCA and 4PBA attenuated MMP9 expression and secretion in the presence of TNFα. Moreover, expression analysis of genes associated with inflammation and autoimmunity identified the c-Fos proto-oncogene as a mediator of ER stress responses in epithelial cells. Substantially less TNFα-induced MMP9 expression occurred when c-Fos signaling was suppressed with a function-blocking antibody. Taken together, these results indicate that activation of ER stress contributes to promote inflammation-mediated proteolytic activity and uncovers a target for restoring tissue homeostasis in ocular autoimmune disease.

## Introduction

About two percent of the human genome is thought to encode proteolytic enzymes responsible for important physiological processes such as development, tissue remodeling and the immune response^1^. The activity of these enzymes must be tightly regulated within an organism to prevent the abnormal degradation of extracellular and cell surface proteins and, consequently, the development or progression of disease. Dysregulation of protease expression has been documented in autoimmune diseases, a group of chronic inflammatory conditions that occur when the immune system turns its antimicrobial defenses against normal components of the body^2^. In the eye, these can be tissue-specific (e.g., Mooren’s ulcerative keratitis), systemic (e.g., cicatricial pemphigoid, Sjögren’s syndrome), or secondary to other autoimmune diseases (e.g., rheumatoid arthritis)^3^.

The loss of self-tolerance in autoimmune disease results in excessive production of cytokines. TNFα is one of the major proinflammatory cytokines contributing to autoimmune pathogenesis due to its direct action on the proliferation and differentiation of immune cells and ability to induce production of additional cytokines^4^. Binding of TNFα to its receptors also induces upregulation of matrix metalloproteinases (MMPs), a family of extracellular endopeptidases long thought to be involved in the remodeling of basement membranes and interstitial connective tissue. In keratinocytes, TNFα exerts a powerful effect on the synthesis of MMP9, a metalloproteinase capable of degrading type IV collagen and long postulated to contribute to the pathogenesis of autoimmune disease^5,6^. Elevated levels of TNFα have been found in epithelial cells of conjunctival tissue from patients with ocular cicatricial pemphigoid^7,8^, but the mechanisms by which this cytokine regulates proteolytic activity during pathological conditions have not been completely elucidated.

The endoplasmic reticulum (ER) is a network of membrane-enclosed tubules and sacs that serve as the first compartment of the secretory pathway in eukaryotic cells^9^. Under normal conditions, a group of molecular chaperones, such as BiP/GRP78, and folding enzymes assist with the assembly of newly synthesized proteins and prevent the misfolding and aggregation of pre-existing proteins. Another group of proteins called the ER-associated degradation (ERAD) pathway assist with the clearance of misfolded proteins. Disruption of ER homeostasis and accumulation of unfolded proteins can overload the ER and induce activation of specific stress signaling pathways, collectively known as the unfolded protein response (UPR)^10^. It involves activation of transcription factors such as XBP1 that direct the expression of chaperones, folding enzymes and components of the ERAD pathway to relieve ER stress and restore homeostasis^11,12^. In pathological conditions, however, prolonged UPR activation initiates cell death. All these pathways have recently gained much attention in autoimmune disease research due to the potential of ER stress proteins to act as autoantigens and their function as quality control factors^13^. Here, we provide evidence indicating that ER stress plays a role in promoting inflammation-mediated proteolytic activity in ocular autoimmune disease and identify the c-Fos proto-oncogene as a key mediator of these events.

## Results

### ER stress is elevated in ocular autoimmune disease

**Figure 1:**
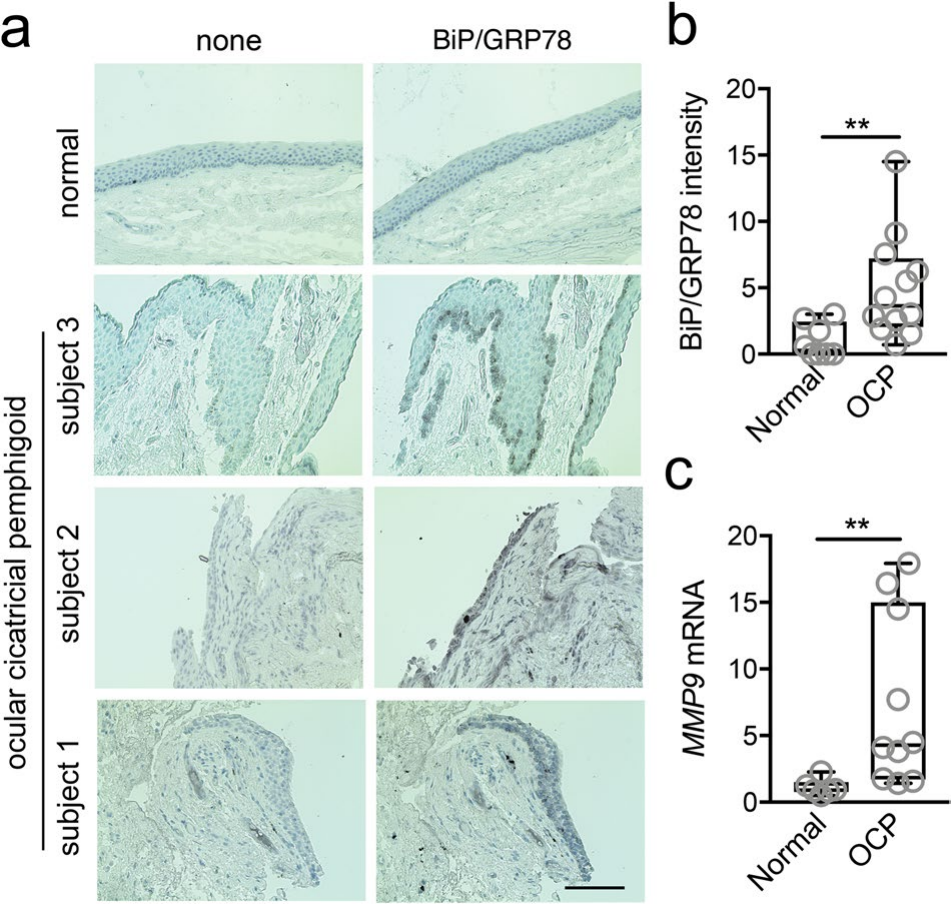
ER stress is elevated in ocular cicatricial pemphigoid. (**a**) BiP/GRP78 staining was analyzed in conjunctival biopsies of three patients with ocular cicatricial pemphigoid (OCP) and three normal subjects by immunohistochemistry. A representative image is shown for normal tissue. Consecutive sections lacking the primary antibody were used as negative controls. Scale bar, 100 μm. (**b**) BiP/GRP78 staining intensity in conjunctival epithelium was measured in at least two different histological areas for each subject using ImageJ. (**c**) Conjunctival epithelium was collected by impression cytology from nine patients with OCP and six normal subjects. The expression of *MMP9* was determined by qPCR. The box and whisker plots show the 25 and 75 percentiles (box), the median, and the minimum and maximum data values (whiskers). Significance was determined using Mann-Whitney test. **p < 0.01.

Past work has evidenced an association between ER stress and autoimmune diseases^14,15^, which at the ocular surface include Sjögren’s syndrome^16^. To expand these findings, we examined tissue from three patients with ocular cicatricial pemphigoid, a type of autoimmune disease that can lead to severe scarring of the conjunctiva and keratopathy^17^. Immunohistochemical analysis demonstrated increased staining of the molecular chaperone BiP/GRP78 in pathological specimens compared to control tissue (Fig. 1a). In these experiments, the antibody bound to the innermost layer of the epithelium in contact with the basement membrane, as well as suprabasal epithelial cells in two of the three pathological specimens. Quantification of the abundance of BiP/GRP78 in the epithelium revealed a 5-fold increase over control tissue (Fig. 1b).

One downstream effect of ER stress following intrinsic or extrinsic challenge is the regulation of processes involved in the remodeling of the extracellular matrix^18,19^. Therefore, we sought to examine the transcriptional levels of *MMP9*, a matrix metalloprotease involved in the degradation of denatured collagens, including basement membrane type IV and anchoring fibril type VII collagens. Analysis of gene expression in conjunctival epithelium revealed a significantly higher content of *MMP9* transcripts in pathological specimens compared to control (Fig. 1c), suggesting a potential association between ER stress and the regulation of the proteolytic microenvironment in ocular autoimmune disease.

### TNFα promotes ER stress at the ocular surface

**Figure 2:**
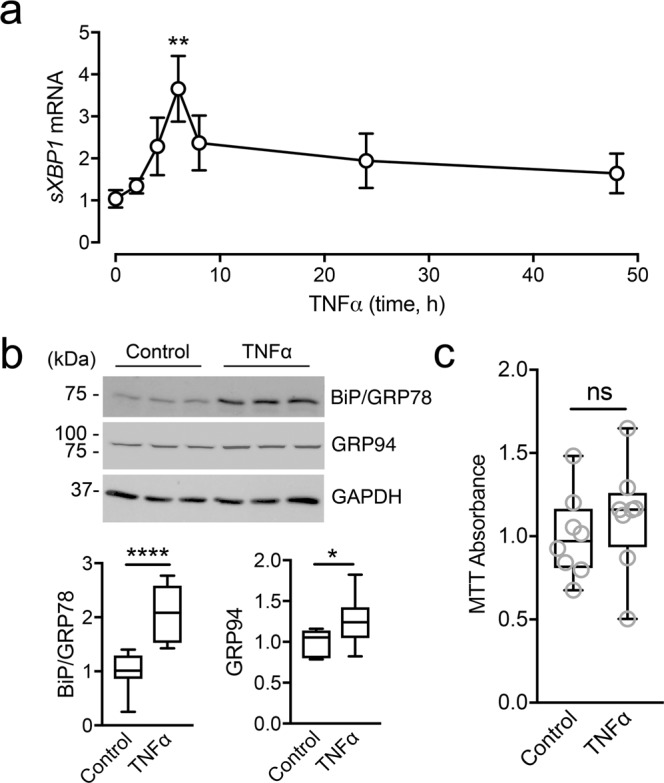
TNFα triggers ER stress in human corneal epithelial cells. (**a**) Multilayered cultures of human corneal epithelial cells were incubated with 40 ng/ml TNFα at different time points. The expression of *sXBP1* was analyzed by qPCR. (**b**) Cell cultures were treated for 48 h with TNFα. Total protein levels of BiP/GRP78 and GRP94 were assessed by immunoblotting. Full-length blots are presented in Supplemental Figure S1. (**c**) Cell viability rates after incubation with TNFα for 48 h were determined by using the MTT assay. Results represent at least three independent experiments. Data in (**a**) represent the mean ± SEM. The box and whisker plots show the 25 and 75 percentiles (box), the median, and the minimum and maximum data values (whiskers). Significance was determined using Kruskal-Wallis with Dunn’s post hoc test (**a**) and Student’s t test (**b**,**c**). *p < 0.05; **p < 0.01; ****p < 0.0001; ns, not significant.

Increased TNFα expression has been found in ocular autoimmune disease. Therefore, in subsequent experiments, we examined the contribution of TNFα to the activation of the UPR in multilayered cultures of corneal epithelial cells. As shown in Fig. 2a, the expression of spliced *XBP1* (*sXBP1*) encoding an active transcription factor that binds to many UPR target genes peaked at 6 h after treatment with TNFα, and progressively decreased thereafter. This event was followed by the biosynthesis of a subset of downstream ER resident chaperones in the UPR that augment ER folding capacity. Exposure to TNFα for 48 h resulted in a significant increase in both BiP/GRP78 and GRP94 (Fig. 2b). In these experiments we did not observe cell toxicity, as measured by cell viability assay (Fig. 2c), suggesting that TNFα under our assay conditions promotes activation of the UPR without triggering cell death mechanisms.

### Pharmacological inhibition of ER stress decreases TNFα-induced *MMP9* expression

**Figure 3:**
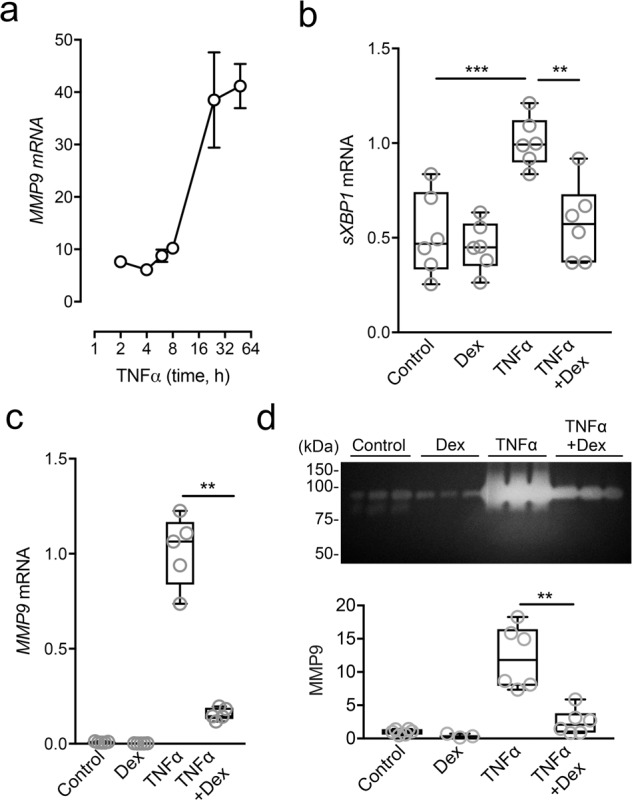
Dexamethasone alleviates ER stress and TNFα-induced *MMP9* expression. (**a**) Multilayered cultures of human corneal epithelial cells were incubated with 40 ng/ml TNFα at different time points. The expression of *MMP9* was analyzed by qPCR. (**b**) The effect of dexamethasone on *sXBP1* expression was measured by qPCR following 6 h incubation with TNFα. (**c**) The effect of dexamethasone on *MMP9* expression was measured by qPCR following 48 h incubation with TNFα. (**d**) Cell culture supernatants in (**c**) were analyzed by gel zymography. Results in (**a**) represent at least three independent experiments. Results in (**b**–**d**) represent two independent experiments performed in triplicate. Data in (**a**) represent the mean ± SEM. The box and whisker plots show the 25 and 75 percentiles (box), the median, and the minimum and maximum data values (whiskers). Significance was determined using one-way ANOVA with Tukey’s post hoc test (**b**) and Mann-Whitney test (**c**,**d**). **p < 0.01; ***p < 0.001. Dex, dexamethasone.

Next, we evaluated whether ER stress was involved in promoting *MMP9* expression and secretion under proinflammatory conditions. TNFα is a potent inducer of MMP9 in human corneal epithelial cells^20^. Consistent with these data, we observed abundant *MMP9* transcripts in our multilayered model of corneal epithelium after cytokine treatment (Fig. 3a). To investigate the role of ER stress in this process, we used dexamethasone, a corticosteroid clinically used to control inflammation and with the ability to suppress the activation of the UPR in epithelial cells^21^. We found that dexamethasone inhibited the expression of *sXBP1* following treatment of the epithelial cultures with TNFα (Fig. 3b). Importantly, dexamethasone significantly impaired the transcription and secretion of MMP9 under proinflammatory conditions (Fig. 3c,d), suggesting that this drug could limit MMP9 production by reducing UPR activation. It should be noted, however, that dexamethasone has pleiotropic effects on multiple signaling pathways that limit its utility as a mechanistic probe.

**Figure 4:**
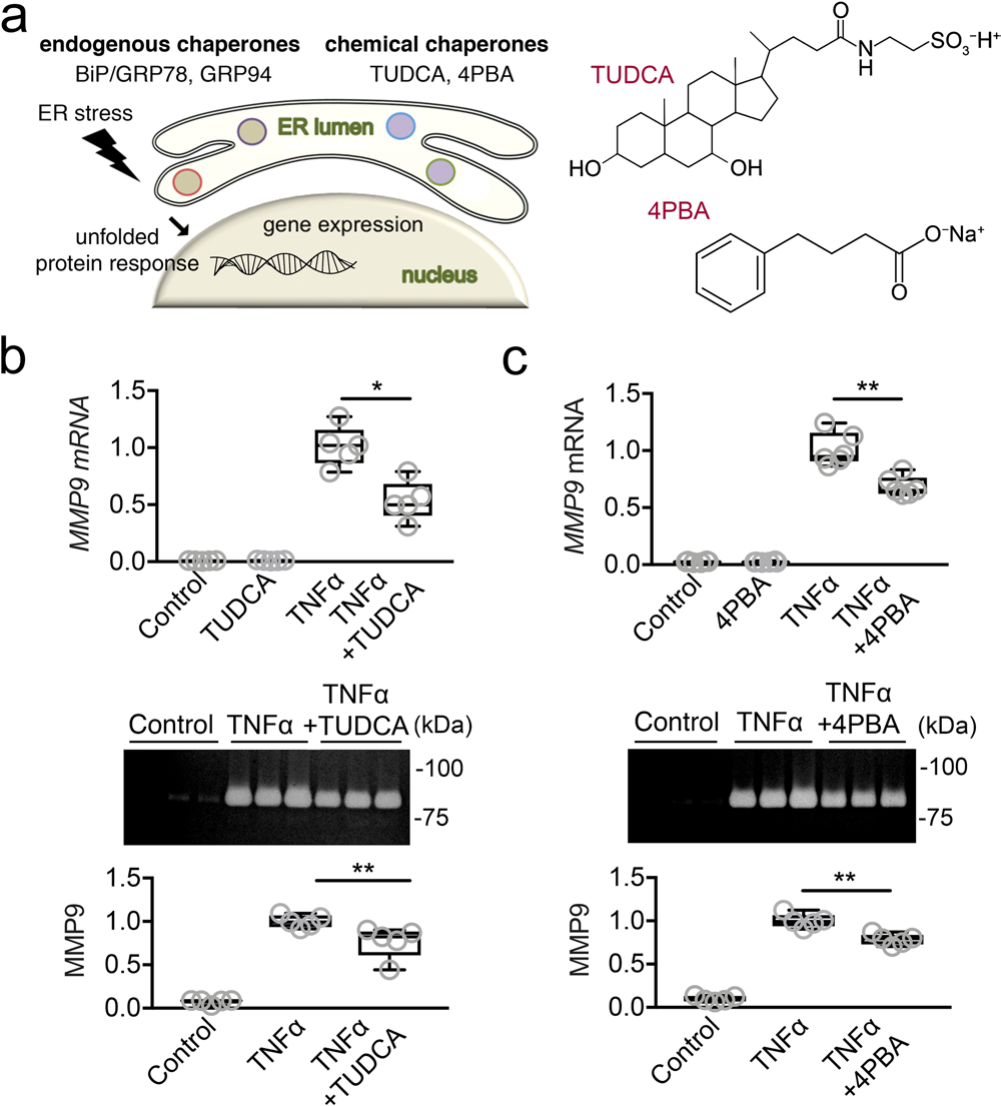
Use of chemical chaperones decreases TNFα-induced *MMP9* expression. (**a**) Diagram showing endogenous and chemical chaperones, which reduce ER stress by stabilizing protein conformation and improving ER folding capacity. (**b**) The effect of TUDCA on *MMP9* expression and secretion into the cell culture media was measured by qPCR and gel zymography, respectively, following 48 h incubation with TNFα. (**c**) The effect of 4PBA on MMP9 expression and secretion was also measured following 48 h incubation with TNFα. Results represent two independent experiments performed in triplicate. The box and whisker plots show the 25 and 75 percentiles (box), the median, and the minimum and maximum data values (whiskers). Significance was determined using Mann-Whitney test. *p < 0.05; **p < 0.01.

Consequently, to further delineate the relationship between ER stress and the production of MMP9 during inflammation, we treated the epithelial cells with two specific inhibitors of ER stress, tauroursodeoxycholic acid (TUDCA) and 4-phenylbutyric acid (4PBA) (Fig. 4a). These are chemical chaperones that reduce ER stress by stabilizing protein conformation and improving ER folding capacity^22^. Incubation with TUDCA and 4PBA following TNFα stimulation significantly reduced the induction of *MMP9* expression as well as its secretion into the cell culture media (Fig. 4b,c). Overall, these results indicate that induction of proteolytic activity in epithelial cells by the proinflammatory cytokine TNFα can be mediated by activation of ER stress.

### ER stress induces *MMP9* expression by promoting *FOS* transcription

**Figure 5:**
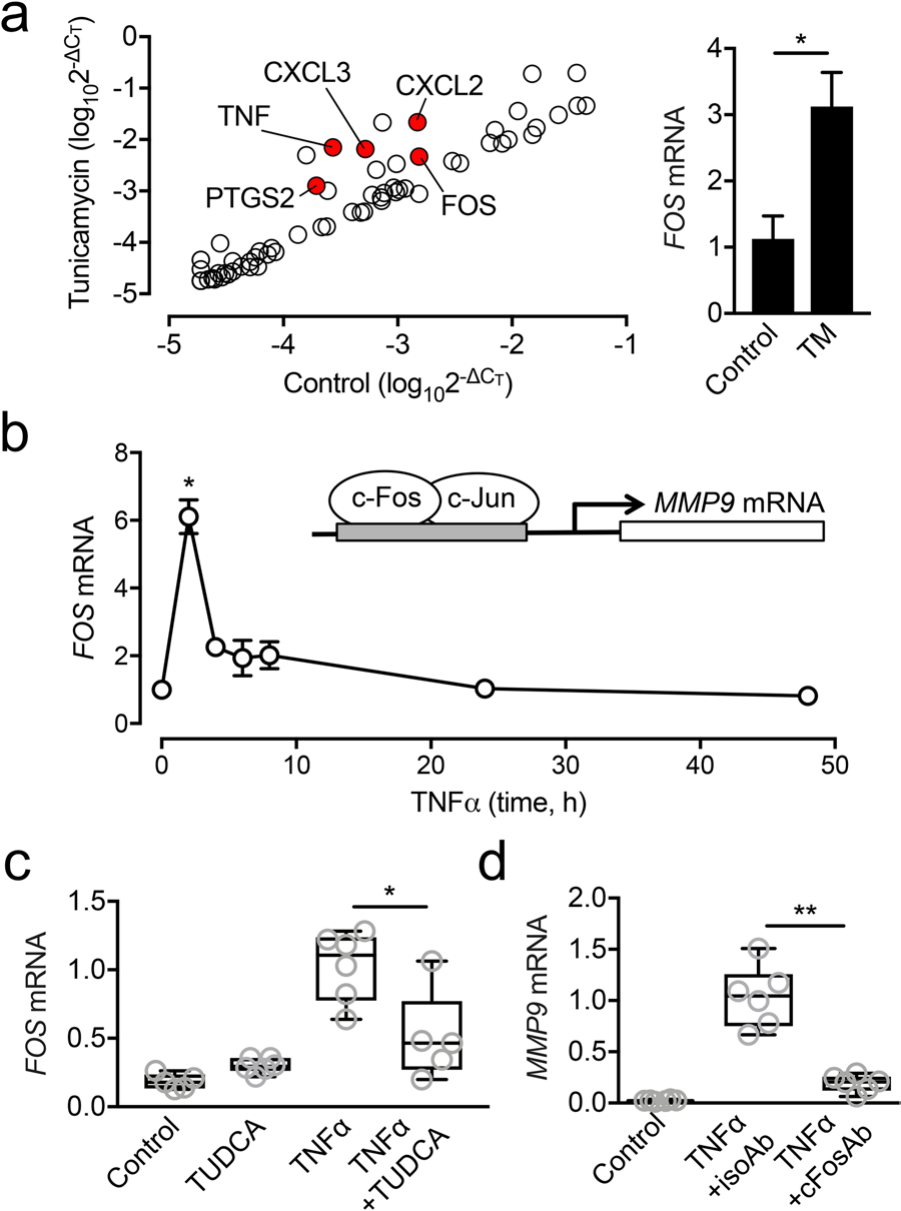
ER stress induces *MMP9* expression by promoting *FOS* transcription. (**a**) Monolayer cultures of human corneal epithelial cells were incubated with 10 µg/ml tunicamycin for 2 h. The relative expression of genes involved in the inflammatory response was evaluated with a human inflammatory response and autoimmunity PCR array. The red dots in the scatter diagram indicate at least 2-fold significant upregulation compared to control. No gene was significantly downregulated. (**b**) Multilayered cultures of human corneal epithelial cells were incubated with 40 ng/ml TNFα at different time points. The expression of *FOS* was analyzed by qPCR. (**c**) The effect of TUDCA on *FOS* expression was measured by qPCR following 2 h incubation with TNFα. (**d**) The effect of a function-blocking antibody to human c-Fos (cFosAb) on *MMP9* expression was measured by qPCR following 48 h incubation with TNFα. An isotype-matched antibody (isoAb) served as control. Results in (**a**,**b**) represent three independent experiments. Results in (**c**,**d**) represent two independent experiments performed in triplicate. Data in (**a**,**b**) represent the mean ± SEM. The box and whisker plots show the 25 and 75 percentiles (box), the median, and the minimum and maximum data values (whiskers). Significance was determined using Student’s t test (**a**), Kruskal-Wallis with Dunn’s post hoc test (**b**) and Mann-Whitney test (**c**,**d**). *p < 0.05; **p < 0.01. TM, tunicamycin.

To gain insight into the mechanism underlying the regulation of *MMP9* expression by ER stress, we treated corneal epithelial cells with tunicamycin, a potent inhibitor of N-glycosylation that disrupts protein maturation and induces ER stress. Changes in the relative expression of genes involved in the inflammatory response were evaluated with a human inflammatory response and autoimmunity PCR array. Scatterplots using a twofold cutoff showed that induction of ER stress evoked a significant increase in *PTGS2*, *TNFα*, *CXCL2*, *CXCL3* and *FOS* (Fig. 5a, Supplemental Table S1). Of interest was *FOS*, which encodes a leucine zipper protein that can dimerize with proteins of the JUN family to form a transcription factor complex known as AP-1. AP-1 has been shown to bind the promoter region of human *MMP9* to regulate its expression^23^.

We continued to examine whether TNFα would promote the transcription of *FOS* in a similar fashion as tunicamycin. In these experiments, we found that the level of expression of *FOS* peaked at 2 h following treatment with TNFα (Fig. 5b). Furthermore, we observed that the expression of *FOS* was reduced by TUDCA (Fig. 5c), implicating ER stress in the expression of components of the AP-1 complex under proinflammatory conditions. Upon further examination, we determined that suppression of c-Fos signaling with a function-blocking antibody significantly impaired *MMP9* expression in our model of TNFα-induced inflammation (Fig. 5d). Together, these data indicate that activation of ER stress during inflammation plays a role in regulating *FOS* expression and the induction of *MMP9*.

## Discussion

Biochemical and functional studies have implicated abnormal proteolytic activity in the pathogenesis of autoimmune disease. These analyses indicate that active MMPs cleave a diverse range of substrates such as cell surface molecules, adhesion proteins and components of the extracellular matrix to influence signaling and tissue remodeling processes^24^. The expression and activity of these enzymes is regulated by a number of factors, and accumulating evidence points to the participation of ER stress proteins in some of these pathways. Here, we report the presence of ER stress at the ocular surface epithelia of patients with ocular cicatricial pemphigoid and demonstrate a role for the UPR in mediating the expression of *MMP9* under proinflammatory conditions. Moreover, we identify the c-Fos proto-oncogene as a transcription factor that governs these events.

Induction of ER stress and subsequent triggering of the adaptive UPR mechanism are processes that have recently gained much attention for their contributions to autoimmune disease. Examples include pemphigus and inflammatory bowel disease, where ER stress and the UPR have been shown to be associated with disease progression and susceptibility, respectively^25,26^. The mechanism by which ER stress contributes to the pathobiology of autoimmune disease appears to reside in the immunogenic nature of misfolded proteins and the function of ER stress proteins as autoantigens^13^. More recent evidence indicates that ER stress proteins also have pathogenic relevance by participating in additional biological processes. An example is the ER chaperone BiP/GRP78, which promotes synovial cell proliferation and angiogenesis through a VEGF-dependent mechanism in rheumatoid arthritis^27^. In our hands, ER stress contributes to the pathobiology of ocular autoimmune disease by regulating the proteolytic microenvironment at the ocular surface. Central to these processes are the proinflammatory cytokines. Addition of TNFα to synoviocytes, hepatocytes or fibrosarcoma cells has been shown to increase the expression of BiP/GRP78 and to activate the UPR^27–29^. Consistent with these results, we also find that TNFα upregulates BiP/GRP78 expression in corneal epithelial cells, supporting the role of proinflammatory cytokines as ER stressors in autoimmune diseases and revealing an important overlap between inflammation and ER stress pathways.

The presence of elevated levels of MMP9 is a common feature in autoimmune conditions such as lupus, Sjögren’s syndrome, multiple sclerosis, and rheumatoid arthritis. In multiple sclerosis, the finding of increased expression of MMP9 in areas of damaged tissue has suggested that this enzyme plays a role in the disruption of vascular basement membranes^30^ whereas in patients with Sjögren’s syndrome it has been linked to the progressive atrophy of the salivary glands and the extensive infiltration by fibrous tissue^31^. MMP9 is also elevated in the tear fluid of patients with ocular cicatricial pemphigoid^32,33^, but its regulation has not been thoroughly investigated so far. Glucocorticoids are a group of steroid compounds with anti-inflammatory properties that can reduce ER stress on epithelial surfaces. In the intestine, dexamethasone alleviates ER stress by enhancing protein folding and the degradation of misfolded proteins^21^. Consistent with these data, we found that dexamethasone reduced the levels of *sXBP1* of corneal epithelial cells under inflammatory stress. Importantly, we observed a concomitant reduction in the expression and secretion of MMP9, suggesting an association between ER stress and proteolytic activity at the ocular surface. The use of the chemical chaperones TUDCA and 4PBA conclusively demonstrated that ER stress supported the actions of TNFα as a regulator of *MMP9* transcription in epithelial cells. Based on these results, it is likely that ER stress contributes to the excessive induction of MMP9 under continued exposure to proinflammatory mediators at the ocular surface.

One question that remains is how ER stress modulates *MMP9* expression under proinflammatory conditions. Using a PCR array we identified *FOS* as a downstream gene targeted for induction by ER stress in corneal epithelial cells. These results were not completely unexpected, since the *MMP9* promoter has multiple functional *cis*-regulatory regions for binding transcriptional factors, including three for AP-1, a heterodimer of c-Jun and c-Fos^23^. Indeed, previous research using the immortalized HaCaT keratinocyte cell line has shown that activation of c-Jun by TNFα is a critical step in the regulation of *MMP9* expression^5,34^. Crucially, these reports point out to mitogen-activated protein kinases (MAPK) as the enzymes responsible for the activation of the AP-1 complex and the expression of *MMP9*. It is well established that there are points of crosstalk between the MAPK signaling cascade and the UPR. MAPK signaling networks are activated in response to extracellular signals that include not only proinflammatory cytokines but also multiple cellular assaults including ER stress^35^. Therefore, it is possible to envision that expression of *MMP9* in epithelial cells is a consequence of the direct action of proinflammatory cytokines on signaling pathways but also the activation of the UPR itself. Future work should focus on determining the relative contribution of each pathway to the expression of proteolytic enzymes during inflammation.

Collectively, our data advance the understanding of the contribution of ER stress to the pathogenesis of ocular autoimmune disease and highlight the complexity of signal transduction pathways activated by proinflammatory cytokines in the regulation of the proteolytic microenvironment. The results also suggest that use of chemical chaperones to alleviate ER stress has potential as a therapeutic strategy for patients with ocular autoimmune disorders.

## Methods

### Human specimens

Conjunctival epithelium was collected by impression cytology from ten eyes of nine patients with ocular cicatricial pemphigoid stage II at the Ocular Surface Center at Campus Bio-Medico University of Rome, Italy. Cells were collected by placing a sterile disc of nitrocellulose membrane on the temporal bulbar conjunctiva using sterile forceps as described^36^. The mean age of the patients was 66.1 ± 13.1 years (range, 44–81 years). Six specimens from six age-matched normal subjects were used as a control group. The mean age of the control group was 62 ± 6.1 years (range, 53–71 years). Exclusion criteria for the control group included history of ocular disease or eye surgery and contact lens wear^37^. Informed consent was obtained from each recruited patient. The study protocol conformed to the ethical guidelines of the 1975 Declaration of Helsinki, and was approved by the Institutional Review Board of Campus Bio-Medico University of Rome. Human conjunctival biopsies stored in paraffin from three normal subjects were obtained as archived material from a previously published study^38^. Conjunctival tissue sections from three patients with ocular cicatricial pemphigoid were obtained from Campus Bio-Medico University of Rome.

### Immunohistochemistry

Six-micron tissue sections of conjunctival epithelium were subjected to routine deparaffinization with xylene and rehydration with graded ethanol. Epitope exposure was performed using citrate buffer (10 mM sodium citrate, 0.05% Tween 20, pH 6.0) at 95 °C for 15 min with subsequent cooling at room temperature for 20 min. Sections were blocked for 1 h in 1% bovine serum albumin and 10% goat serum in phosphate buffered saline and incubated with rabbit anti-human BiP/GRP78 antibody (C50B12; 1:200; Cell Signaling Technology) in 1% bovine serum albumin and 5% goat serum in phosphate buffered saline at 4 °C overnight. Tissues were exposed to 0.3% hydrogen peroxide in phosphate buffered saline for 10 min to quench endogenous peroxidase activity, followed by sequential application of a secondary anti-rabbit antibody (VWR) conjugated to biotin for 45 min, and the HRP-streptavidin complex (Vector Laboratories) for 30 min at room temperature. Immunoreactivity was detected using SigmaFast™ DAB with metal enhancer (Sigma-Aldrich) and nuclei counterstained with hematoxylin QS (Vector Laboratories). Coverslips were mounted using aqueous mounting medium, and staining was visualized by light microscopy. Incubation with primary antibody was routinely omitted in control experiments to demonstrate lack of endogenous peroxidase activity. Positive staining was analyzed by hand-outlining areas of conjunctival epithelium in ImageJ (National Institutes of Health) and measuring the density of brown staining per area using the H DAB vector. Mean staining per area in sections treated without the primary antibody was subtracted as background.

### Cell culture

Cultures of telomerase-immortalized human corneal epithelial cells were grown as previously reported^36^. Briefly, cells were plated at a seeding density of 1 × 10^5^ cells/cm^2^ and maintained in keratinocyte serum-free medium (Thermo Fisher Scientific) supplemented with 0.3 mM CaCl_2_, 25 µg/ml bovine pituitary extract, 0.2 ng/ml epidermal growth factor, and 1% penicillin/streptomycin until confluence. Thereafter, cells were grown in Dulbecco’s Modified Eagle’s Medium (DMEM)/F12 supplemented with 10% calf serum, 10 ng/ml epidermal growth factor, and 1% penicillin/streptomycin for 7 days to promote stratification and differentiation. Where indicated, multilayered cultures were serum-starved for 1 h and incubated with TNFα (40 ng/ml; PeproTech) in serum-free DMEM/F12 in the presence and absence of dexamethasone (10 µg/ml; Sigma-Aldrich), 4PBA (2.5 mM; Sigma-Aldrich), TUDCA (1 mM; Merck Millipore), a blocking antibody to human c-Fos (6–2H-2F; 2 µg/ml, Santa Cruz Biotechnology) or mouse IgG control (sc-2025; 2 µg/ml, Santa Cruz Biotechnology). Monolayer cultures were treated with tunicamycin (10 µg/ml; Sigma-Aldrich) or DMSO control.

### Immunoblotting

Cells were lysed in RIPA buffer supplemented with Complete™ EDTA-free Protease Inhibitor Cocktail (Roche Diagnostics) as described^39^. After homogenization with a pellet pestle, the cell extracts were centrifuged at 17,115 × g for 45 min at 4 °C, and the protein concentration of the supernatant determined using the Pierce BCA™ Protein Assay Kit (Thermo Fisher Scientific). Proteins were separated by SDS-PAGE (10% resolving gel) and electroblotted onto nitrocellulose membranes. Nonspecific binding to the nitrocellulose was blocked by incubation with 5% nonfat milk in 0.1% Tween 20 in Tris-buffered saline at room temperature for 1 h. Membranes were incubated with primary antibodies to BiP/GRP78 (1:1,000; C50B12, Cell Signaling Technology) and GRP94 (1:1,000; #2104; Cell Signaling Technology) in blocking buffer overnight at 4 °C. GAPDH (1:3,000; FL-335; Santa Cruz Biotechnology) served as a sample loading control. Membranes were then incubated with the appropriate secondary antibodies coupled to horseradish peroxidase (1:5,000; Santa Cruz Biotechnology) for 1 h at room temperature. Peroxidase activity was visualized using chemiluminescence. Densitometry was performed using ImageJ software.

### Gel zymography

Cell culture medium from 12-well plates was collected and centrifuged at 1,150 × g for 5 min to remove cells and cellular debris as described^40^. The supernatant (15 μl) was mixed with non-reducing loading buffer (50 mM Tris-HCl pH 6.8, 10% glycerol, 1% SDS, and 0.01% Bromophenol Blue) and resolved on 7.5% SDS-PAGE gels containing 1 mg/ml gelatin (bovine skin type B). Gels were then incubated in 50 mM Tris containing 5 mM CaCl_2_ and 2.5% Triton X-100 for two h at room temperature, refreshing the buffer every 30 min. After washing with distilled water, gels were incubated in collagenase buffer (50 mM Tris-HCl pH 7.6, 5 mM CaCl_2_) for 24 h at 37 °C followed by staining in Coomassie Brilliant Blue solution (40% methanol, 10% acetic acid, 0.025% Coomassie Brilliant Blue R-250). Gels were then washed in distilled water for 2 h and photographed. Gelatinase activity was quantified using ImageJ software.

### RNA isolation and cDNA synthesis

Total RNA was extracted from impression cytology samples and cell cultures using a Qiagen RNeasy Plus Mini Kit (Qiagen) according to the manufacturer’s protocol. Up to 1 μg total RNA was used for cDNA synthesis (iScript™ cDNA Synthesis; Bio-Rad).

### qPCR

Gene expression levels were detected by quantitative real-time PCR using the KAPA SYBR® FAST qPCR kit (Kapa Biosystems) in a Mastercycler ep realplex thermal cycler (Eppendorf). Primer sequences for *sXBP1* (forward 5′-CTGAGTCCGAATCAGGTGCAG-3′; reverse 5′-ATCCATGGGGAGATGTTCTGG-3′) mRNA have been previously published^41^. Primer sequences for *MMP9* (Unique Assay ID qHsaCID0011597)*, FOS* (Unique Assay ID qHsaCED0046695) and *GAPDH* (Unique Assay ID qHsaCED0038674) mRNA were obtained from Bio-Rad. The following parameters were used: 2 min at 95 °C, followed by 40 cycles of 5 seconds at 95 °C and 30 seconds at 60 °C. All samples were normalized using GAPDH housekeeping gene expression. The comparative ΔΔC_T_ method was used for relative quantitation of the number of transcripts^37^. No template controls were run in each assay to confirm lack of DNA contamination in the reagents used for amplification.

### Human inflammatory response and autoimmunity PCR array

The analysis of 84 genes encoding for inflammatory cytokines and chemokines and their receptors was carried out using a human inflammatory response and autoimmunity PCR array (PAHS-077Z; RT2 Profiler PCR array, SABiosciences) according to the manufacturer’s instructions. Expression values were corrected for the housekeeping gene GAPDH. The ΔC_T_ method was used for relative quantitation of the number of transcripts.

### MTT assay

Cell viability was assessed by means of the 3-(4,5-dimethylthiazol-2-yl)-2,5-diphenyltetrazolium bromide (MTT) assay following the manufacturer’s instructions (Molecular Probes). Briefly, cultures were incubated with a 1.2 mM MTT solution at 37 °C for 4 h as described^37^. The absorbance values of blue formazan were determined at 540 nm. Cell viability was expressed as MTT uptake in treated cells normalized to untreated cells.

### Statistical analyses

All statistical analyses were performed using Prism 7 (GraphPad Software) for Mac OS X.

## Supplementary information

Supplementary Information.
