# Supplementary material for: Endoplasmic reticulum stress promotes inflammation-mediated proteolytic activity at the ocular surface
Source: Sci Rep. 2020 Feb 10;10:2216. doi: 10.1038/s41598-020-59237-3 (PMC7010695; doi:10.1038/s41598-020-59237-3)
Supplement: Supplementary file 1 — Supplementary Information. [file 41598_2020_59237_MOESM1_ESM.docx]

**Endoplasmic reticulum stress promotes inflammation-mediated proteolytic activity at the ocular surface**

*Ashley M. Woodward^1^, Antonio Di Zazzo^2^, Stefano Bonini^2^, Pablo Argüeso^1,^**

**SUPPLEMETAL MATERIAL**

**Supplemental Figure S1.** Full-length gels/blots used in figures.

Figure 2b (BiP/GRP78)

*
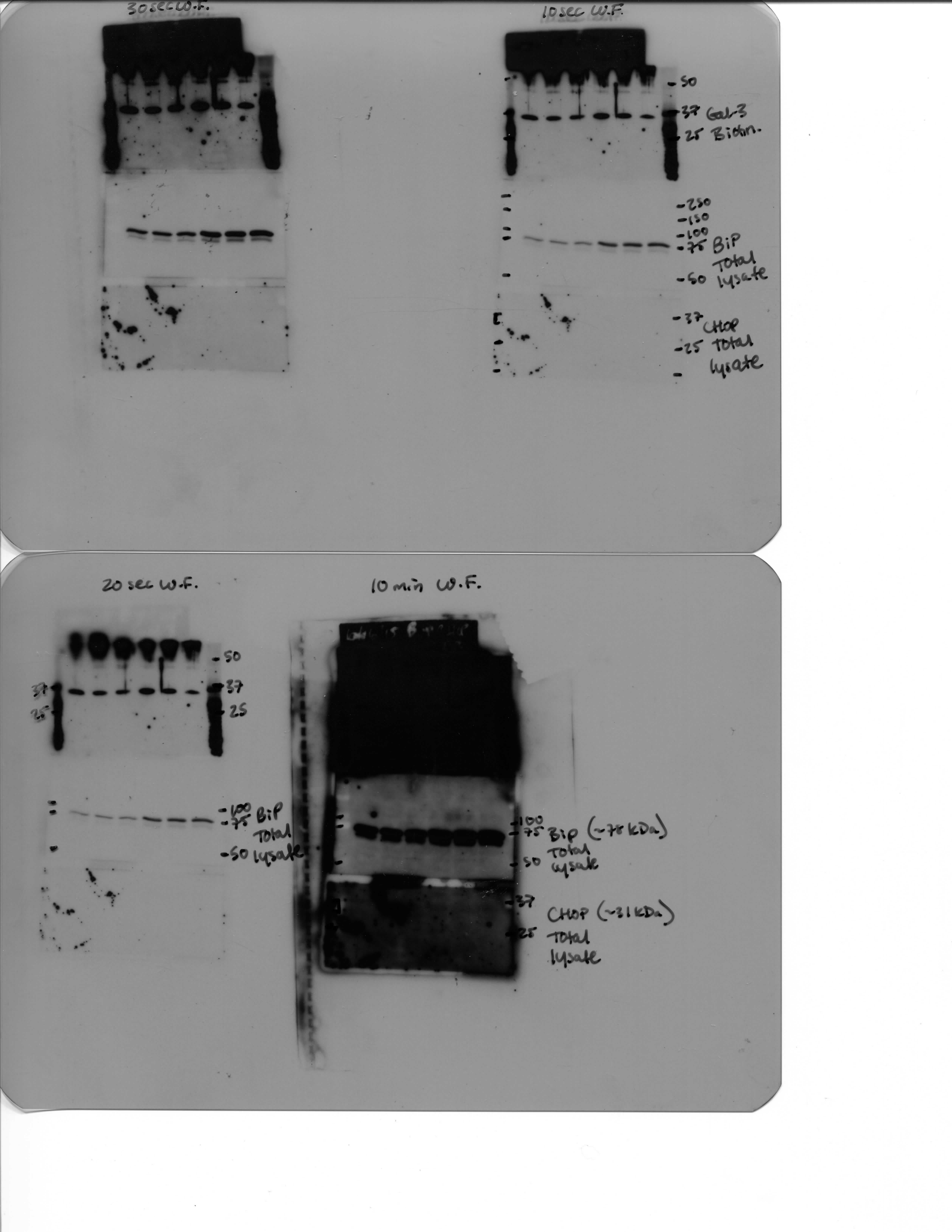
*

Figure 2b (GRP94)

*
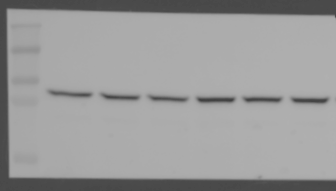
*

Figure 2b (GAPDH)


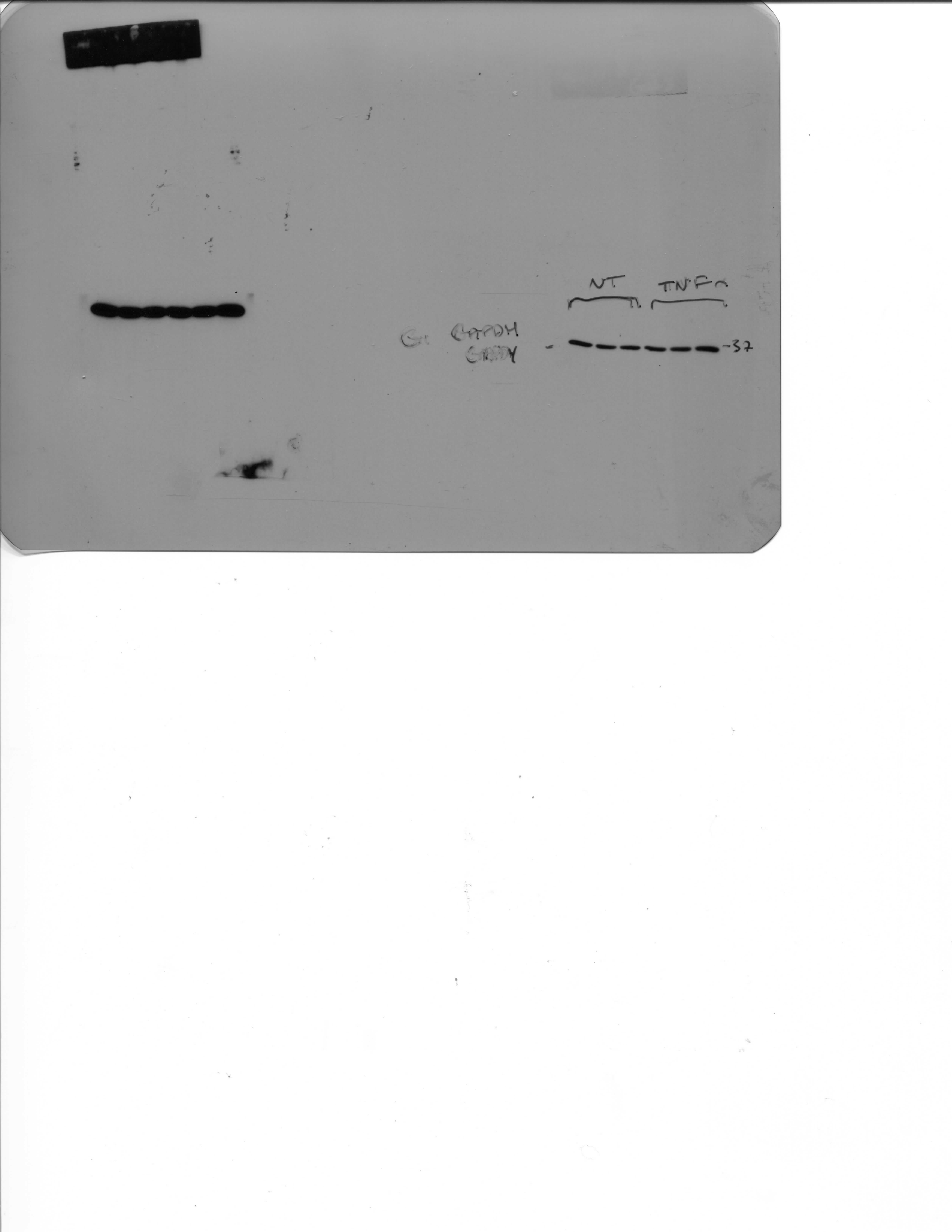


Figure 3d


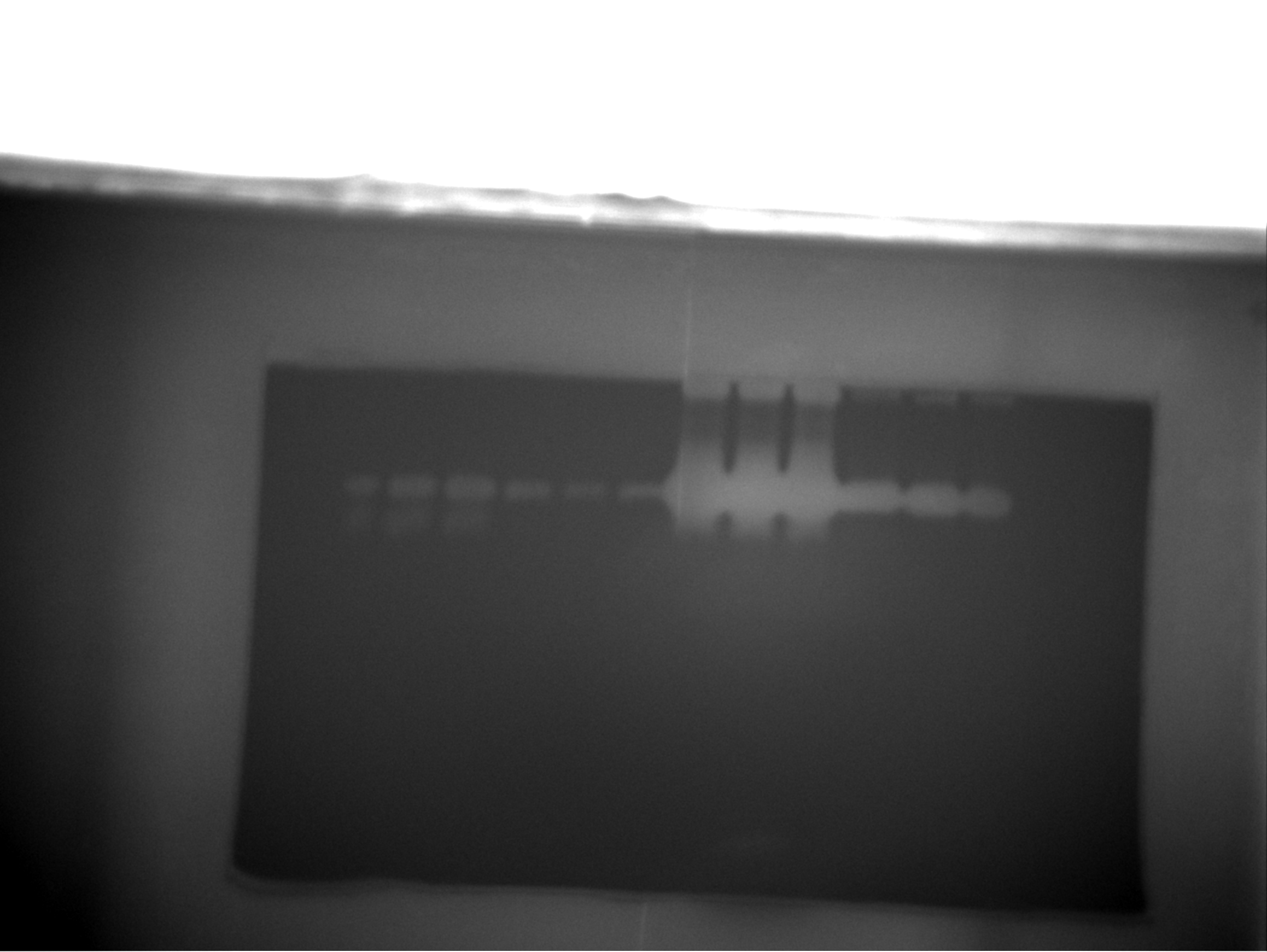


Figure 4b


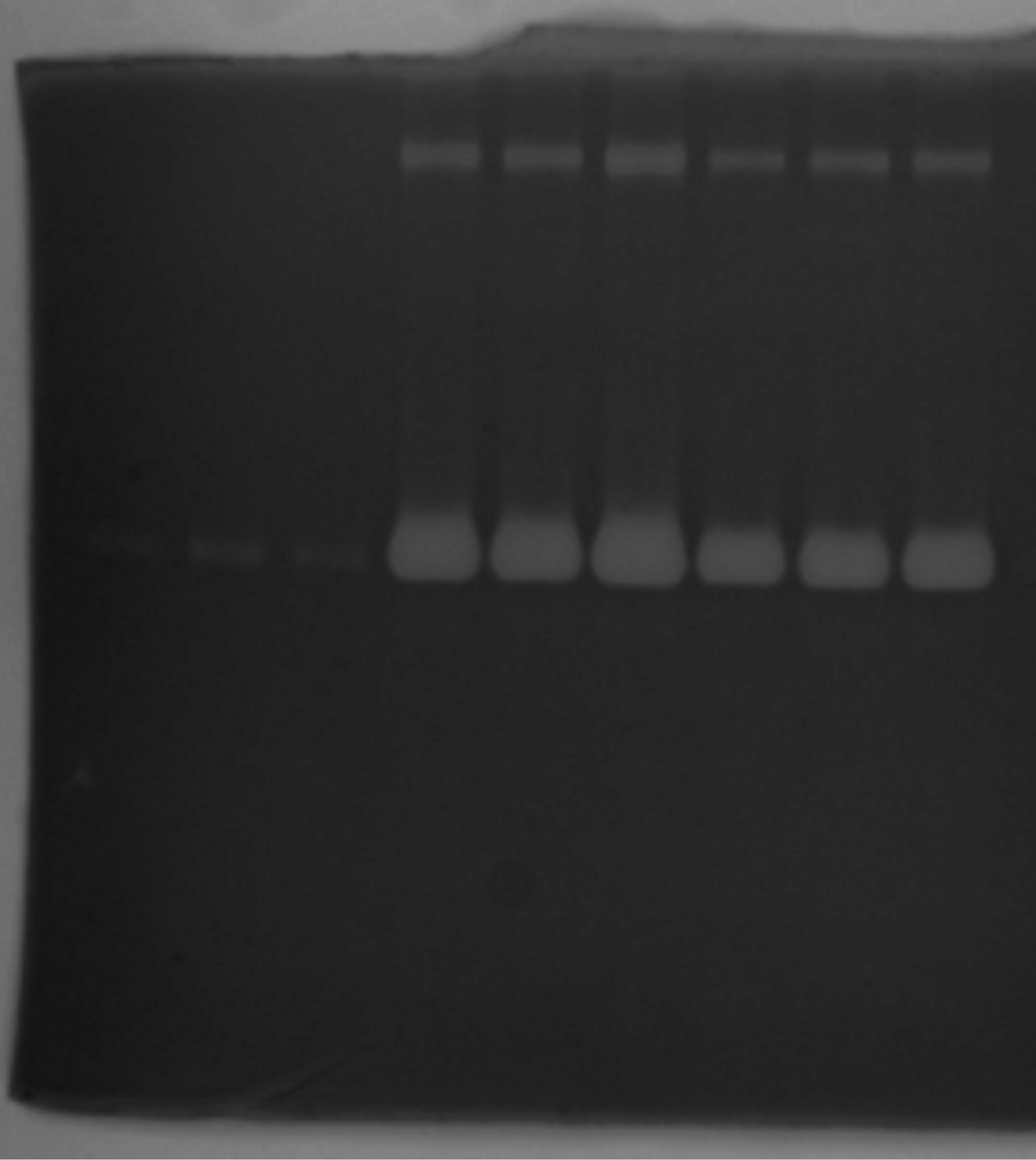


Figure 4c

**
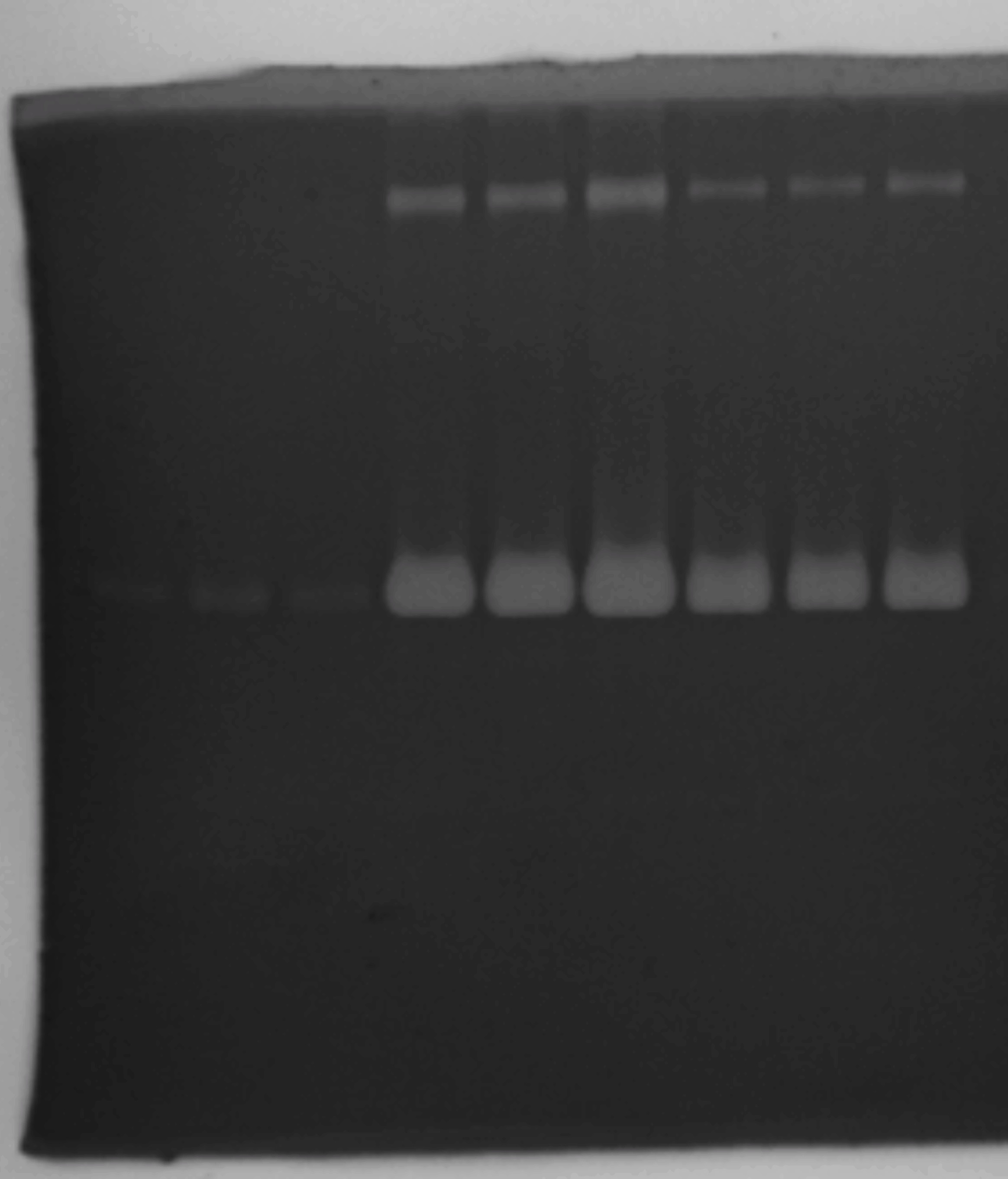
**

**Supplemental Table S1**. Relative expression of genes responsible for inflammation and autoimmunity in human corneal epithelial cells. Cell cultures were incubated with 10 µg/ml tunicamycin for 2 h. Values in bold indicate statistically significant changes in gene expression compared to untreated cells. High ΔC_T_ values reflect low amplification efficiency.

| **Gene Symbol** | **RefSeq*** | **Magnitude of Expression (ΔC_T_)** | **Fold Change** | ***p* value** |
| --- | --- | --- | --- | --- |
| *BCL6* | NM_001706 | 9.35 | 0.57 | 0.249 |
| *C3* | NM_000064 | 10.09 | 1.26 | 0.952 |
| *C3AR1* | NM_004054 | 15.05 | 0.71 | 0.476 |
| *CCL11* | NM_002986 | 15.69 | 0.97 | 0.765 |
| *CCL13* | NM_005408 | 15.69 | 0.97 | 0.765 |
| *CCL16* | NM_004590 | 15.69 | 0.97 | 0.765 |
| *CCL17* | NM_002987 | 15.69 | 0.97 | 0.765 |
| *CCL19* | NM_006274 | 15.69 | 0.97 | 0.765 |
| *CCL2* | NM_002982 | 15.69 | 2.41 | 0.179 |
| *CCL21* | NM_002989 | 15.69 | 0.97 | 0.765 |
| *CCL22* | NM_002990 | 15.18 | 0.94 | 0.719 |
| *CCL23* | NM_005064 | 15.69 | 0.97 | 0.765 |
| *CCL24* | NM_002991 | 15.69 | 0.97 | 0.765 |
| *CCL3* | NM_002983 | 15.69 | 0.97 | 0.765 |
| *CCL4* | NM_002984 | 15.69 | 2.41 | 0.299 |
| *CCL5* | NM_002985 | 10.36 | 1.20 | 0.988 |
| *CCL7* | NM_006273 | 15.69 | 0.97 | 0.765 |
| *CCL8* | NM_005623 | 15.69 | 0.97 | 0.765 |
| *CCR1* | NM_001295 | 14.77 | 0.76 | 0.104 |
| *CCR2* | NM_001123396 | 15.69 | 0.97 | 0.765 |
| *CCR3* | NM_001837 | 14.52 | 0.80 | 0.228 |
| *CCR4* | NM_005508 | 14.01 | 0.57 | 0.373 |
| *CCR7* | NM_001838 | 14.99 | 0.82 | 0.606 |
| *CD14* | NM_000591 | 11.03 | 0.80 | 0.711 |
| *CD40* | NM_001250 | 8.16 | 0.99 | 0.804 |
| *CD40LG* | NM_000074 | 15.35 | 0.81 | 0.494 |
| *CEBPB* | NM_005194 | 6.47 | 3.18 | 0.123 |
| *CRP* | NM_000567 | 15.69 | 0.97 | 0.765 |
| *CSF1* | NM_000757 | 10.71 | 1.41 | 0.819 |
| *CXCL1* | NM_001511 | 12.62 | 31.20 | 0.052 |
| *CXCL10* | NM_001565 | 10.59 | 3.98 | 0.647 |
| ***CXCL2*** | **NM_002089** | **9.39** | **14.59** | **0.041** |
| ***CXCL3*** | **NM_002090** | **10.90** | **12.55** | **0.006** |
| *CXCL5* | NM_002994 | 13.74 | 0.79 | 0.483 |
| *CXCL6* | NM_002993 | 15.69 | 0.97 | 0.765 |
| *CXCL9* | NM_002416 | 15.31 | 0.76 | 0.497 |
| *CXCR1* | NM_000634 | 14.11 | 0.90 | 0.661 |
| *CXCR2* | NM_001557 | 12.03 | 0.85 | 0.648 |
| *CXCR4* | NM_003467 | 15.69 | 0.97 | 0.765 |
| *FASLG* | NM_000639 | 14.27 | 0.66 | 0.212 |
| ***FOS*** | **NM_005252** | **9.34** | **3.03** | **0.033** |
| *IFNG* | NM_000619 | 15.69 | 0.97 | 0.765 |
| *IL10* | NM_000572 | 15.69 | 0.97 | 0.765 |
| *IL10RB* | NM_000628 | 6.75 | 1.08 | 0.988 |
| *IL15* | NM_000585 | 10.45 | 1.05 | 0.792 |
| *IL17A* | NM_002190 | 15.69 | 0.97 | 0.765 |
| *IL18* | NM_001562 | 4.49 | 1.02 | 0.835 |
| *IL1A* | NM_000575 | 6.06 | 12.76 | 0.055 |
| *IL1B* | NM_000576 | 4.77 | 5.40 | 0.066 |
| *IL1R1* | NM_000877 | 9.75 | 0.96 | 0.755 |
| *IL1RAP* | NM_002182 | 5.93 | 1.03 | 0.794 |
| *IL1RN* | NM_000577 | 4.74 | 1.22 | 0.672 |
| *IL22* | NM_020525 | 14.90 | 0.73 | 0.123 |
| *IL23A* | NM_016584 | 12.00 | 4.12 | 0.157 |
| *IL23R* | NM_144701 | 15.69 | 0.97 | 0.765 |
| *IL5* | NM_000879 | 12.18 | 0.92 | 0.867 |
| *IL6* | NM_000600 | 15.11 | 3.41 | 0.259 |
| *IL6R* | NM_000565 | 9.93 | 1.02 | 0.885 |
| *CXCL8* | NM_000584 | 10.41 | 29.04 | 0.111 |
| *IL9* | NM_000590 | 15.69 | 0.97 | 0.765 |
| *ITGB2* | NM_000211 | 12.86 | 1.05 | 0.735 |
| *KNG1* | NM_000893 | 15.69 | 0.97 | 0.765 |
| *LTA* | NM_000595 | 13.53 | 0.76 | 0.547 |
| *LTB* | NM_002341 | 10.00 | 3.43 | 0.945 |
| *LY96* | NM_015364 | 14.27 | 0.82 | 0.645 |
| *MYD88* | NM_002468 | 5.29 | 1.18 | 0.783 |
| *NFKB1* | NM_003998 | 7.14 | 2.17 | 0.121 |
| *NOS2* | NM_000625 | 14.75 | 1.19 | 0.783 |
| *NR3C1* | NM_000176 | 6.05 | 0.82 | 0.506 |
| ***PTGS2*** | **NM_000963** | **12.32** | **6.53** | **0.004** |
| *RIPK2* | NM_003821 | 7.29 | 1.34 | 0.960 |
| *SELE* | NM_000450 | 15.69 | 1.57 | 0.486 |
| *TIRAP* | NM_001039661 | 10.44 | 0.90 | 0.726 |
| *TLR1* | NM_003263 | 13.63 | 0.99 | 0.746 |
| *TLR2* | NM_003264 | 8.38 | 1.27 | 0.648 |
| *TLR3* | NM_003265 | 10.02 | 0.99 | 0.916 |
| *TLR4* | NM_138554 | 13.97 | 1.08 | 0.767 |
| *TLR5* | NM_003268 | 11.28 | 0.97 | 0.914 |
| *TLR6* | NM_006068 | 10.94 | 0.78 | 0.550 |
| *TLR7* | NM_016562 | 15.69 | 0.97 | 0.765 |
| *TLR9* | NM_017442 | 15.68 | 0.96 | 0.751 |
| ***TNF*** | **NM_000594** | **11.85** | **25.87** | **0.031** |
| *TNFSF14* | NM_003807 | 15.44 | 0.86 | 0.600 |
| *TOLLIP* | NM_019009 | 6.93 | 1.02 | 0.900 |

*Reference sequence database at NCBI (https://www.ncbi.nlm.nih.gov/refseq/)
